# How Doctors Are Made

**Published:** 1891-09-12

**Authors:** 


### How Doctors are Made.

An anxious parent writes to us about his son, whom he wishes to educate for the medical profession; and as his letter expresses the uncertainties and anxieties of many other parents, we shall take the liberty of quoting a few passages from it, and of placing our own experience and knowledge at the disposal of those who may be needing guidance on this particular question. "I have a son," says Mr. ——, "about eighteen years of age. He wishes to be educated in the medical profession. He has taken the preliminary examinations at the College of Preceptors. . . . Some of my friends recommend me to send him to the Liverpool or Manchester Medical School, others to the London University, and others, again, to Cambridge, entering him at Caius or Selwyn. Would you give me your advice? I may say that I am inclined to the latter course, as the young man would be in college and under some restraint, as well as having my nephew to look after him. But I do not know the cost of the respective plans or places, nor the time which must expire before a practising qualification can be obtained. My means are limited; but I want to do the best possible for my son, and also to put as much wholesome restraint on him as I can. He has been carefully trained, and up to the present time tries to do the right. The cost, time to be occupied, and quality of education are the main points with me; of course, including also the 'tone' of the various schools."

The parent who puts his son into medicine may give him one of two kinds of education. He may, in the first place, secure for him a training which will make him a general practitioner, and keep him one for ever. He may, on the other hand, help him to such an education and position as will both fit him for general practice and give him the culture and status which will enable him, should opportunity offer, to rise to the higher ranks of the profession. Every far-seeing parent who can by any means afford to choose the latter alternative will certainly do so. It is hardly too much to say that no young man should enter the medical profession with the deliberate purpose of acquiring the minimum of education, and the cheapest and poorest qualifications, and of remaining a full private in the medical army all his life. There must be full privates in the army of medicine, as in other armies; but every one of them should have at least so much education as will enable him, if he possesses the requisite capacity, to rise to the field marshal's level. There is nothing more conducive to individual advantage or to general medical progress than the stimulus furnished by a hopeful ambition.

Our anxious parent admits that he is a man of limited means. Does he suppose that the majority of physicians and surgeons are the sons of men of unlimited means? It is to be presumed that he can spare something for his son's education—say from five hundred to a thousand pounds. If he can manage this he may, by its judicious expenditure, give his son the best medical education which Great Britain has to offer.

At the outset it must be determined whether a hospital or a university shall have the preference. For our part we pronounce for the university, and for an English university if the time and money can be spared. Let this fact be well considered at this point, that practically all medical students must now spend at least five years in professional study after passing their preliminary examinations, and that by devoting a single additional year to college and hospital work the Oxford or Cambridge degree in medicine may be taken. Cambridge is the university selected by our correspondent. In order to take a degree in medicine at Cambridge, and this also is true of Oxford, a man must first take his degree in arts, his B.A. For the B.A., a college residence of three years is compulsory. But then a man of average industry can easily take one or two years of medical study at the same time that he is in residence for his arts degree, that is to say, the two courses of study can run concurrently to a certain extent. A fairly diligent man can take both his B.A. and his M.B. in six years, and the cost of the two, including books, clothes, and everything else essential for a student's well-being, need not exceed one hundred and fifty pounds a year. If the student be economical and industrious, and have a capacity for "ways and means," it may be a good deal less than this, say, a hundred and twenty pounds a year. Any man who begins his professional career with a Cambridge degree may be said to commence with an officer's commission in his pocket.

What has been said of Cambridge applies to Oxford, and, therefore, nothing more need be added on the subject of the two great English Universities. There are, however, other institutions in England which confer University degrees, though they cannot be said to give University training. Chief among these is the London University, so-called. It is not a University in the generally accepted sense of that term, in that it has no colleges, no teachers, and no students. It is an examining and degree conferring body, a kind of conglomerate of school inspectors. Our correspondent speaks of sending his son to the London University for education. There is no London University to which anybody can send his son for education. The man who wishes to take a medical degree at the Examining University of London must study at one or other of the London or provincial hospitals. In that case it is evident that he will not have a University education, but a hospital education. He will be bred among doctors pure and simple, and not among men of all faculties. That is the objection which is commonly urged against the London University and its graduates; the objection, namely, that the University is no University in that it does not teach, and that its graduates are not University men in that they have had no University education. It is known and admitted that the examinations for the London University degrees in medicine are as severe as those at Oxford, Cambridge, and Edinburgh, and more severe than those carried out by some other bodies that give medical qualifications. The status, that is to say, the purely medical status, of the London University degree, ranks therefore with the highest; but its social status is decidedly lower than are the degrees of Oxford and Cambridge. The London University degree can be obtained in five or six years, and the economical student need not spend more than from a hundred to a hundred and thirty pounds a-year in obtaining it.

The Scotch Universities give an excellent medical training, at a comparatively moderate cost, and in the case of Edinburgh more especially, a degree in medicine which has always ranked with the highest. Taking Edinburgh as the most desirable of the Scotch Universities for an English student of good social position, we will briefly indicate its most characteristic features. The Edinburgh University, to begin with, has the largest medical school in Great Britain, perhaps in the world. It draws its students not only from England and Scotland, but also from the Colonies and foreign countries. It has a highly paid staff of eminent teachers. It is a university with the faculties of arts, law, and theology, as well as of medicine. It gives, therefore, a University education. It does not require a degree in arts of its medical graduates, as Oxford and Cambridge do; but, like the London University, it holds a special matriculation examination for its own students. The period of study at the Edinburgh University is from five to six years, and the cost to an economical student need not be more than from a hundred to a hundred and thirty pounds a-year. The Edinburgh University is not to be confounded with the Edinburgh Extramural School of Medicine. The latter is on a lower plane altogether in point both of education and of qualifications conferred. It must be admitted also that it is on a much lower level in point of expensiveness.

The principal universities which give degrees in medicine in England and Scotland have now been indicated. It is proper also to mention the Victoria University at Manchester and the University of Durham in England, and also the Universities of Glasgow and Aberdeen in Scotland. Each of these gives a good medical training and a respectable degree at a moderate cost. The Dublin University in Ireland is also an institution of note and name. But as very few Englishmen go to Ireland for medical education it is hardly necessary to give further details here.

The bulk of general practitioners of medicine do not enter for university degrees at all. They are content with such diplomas as M.R.C.S. England, L.R.C.P. London, L.R.C.P. Edinburgh, L.R.C.S. Edinburgh, and even in some cases with the humble but useful distinction of L.S.A.

Turning now for a moment to medical schools, rather than to universities, it is to be understood that the great hospitals of London—Guy's, St. Bartholomew's, St. Thomas's, the London Hospital, University College Hospital, St. Mary's, and others; and also the provincial hospitals, such as Liverpool, Manchester, Birmingham, and Leeds, prepare students in whole or in part for all the degrees and diplomas, whether given by Universities or by Colleges of Physicians and Surgeons. If a man enters for the London University degree, he must take all his qualifying classes at one or other of the great hospitals in London, or the provinces, or Scotland. If he enters for Cambridge or Oxford, he must, nevertheless, take some of his qualifying classes at one or more of the great hospitals. At Edinburgh and the other Scotch Universities he may complete his curriculum without leaving the University; but even at those institutions he is permitted, if he wishes, to take half of his curriculum at one or more of the great London or provincial hospitals.

This article has grown to a somewhat undue length. It might have been made ten times longer without exhausting all the useful information which could be given on the subject. We close with one final word. It is this—that every young man should be put into the way of getting a good education and a respectable qualification. The first thing to do is to choose the degree that shall be aimed at, then to write to the University or Royal College which gives the degree and ask for the regulations and conditions under which it is to be obtained; and finally to decide upon the college or hospital where detailed study is to be carried out. Every college and hospital in the kingdom is glad to send a prospectus containing all needful information to anxious parents and sons who may be contemplating the manufacture of new doctors.

